# Editorial: Reviews in psychiatry 2022: personality disorders

**DOI:** 10.3389/fpsyt.2023.1279335

**Published:** 2023-09-04

**Authors:** Bo Bach, Massimiliano Beghi

**Affiliations:** ^1^Center for Personality Disorder Research (CPDR), Psychiatric Research Unit, Slagelse Psychiatric Hospital, Slagelse, Denmark; ^2^Department of Psychology, University of Copenhagen, Copenhagen, Denmark; ^3^Department of Mental Health and Addictions, Azienda Unità Sanitaria Locale (AUSL) della Romagna, Cesena, Italy

**Keywords:** personality disorder, dimensional models, diagnosis, borderline, narcissism, musculoskeletal disorder, ICD-11, AMPD

## Introduction

In this editorial, we provide an overview and discussion of key points from the nine papers published in this 2022 Research Topic entitled “*Reviews in psychiatry 2022: personality disorders*.” The overview is thematically organized by Research Topic.

## New perspectives on the personality disorder diagnosis

Gutiérrez and Valdesoiro provide a review of proposals for understanding personality disorders (PD) from the perspective of evolutionary theory. The authors highlight that personality differences are ubiquitous in nature, from insects to higher primates and humans. They stress that from such an evolutionary perspective, we can truly explain why harmful personalities exist at all, and why they remain over time. We consider this perspective informative and consistent with the ICD-11 and alternative model of personality disorders (AMPD) frameworks of personality functioning and what it actually means to be human from a psychological perspective (1, 2).

Monaghan and Bizumic give an overview of challenges and opportunities related to exchanging traditional categories of PD with dimensional models. The authors point out the need for ongoing development of a broader array of measurement methods (e.g., multimethod assessments, influence of social desirability, and the potential of using opposite poles of dysfunction) and for a wider communication and training in dimensional approaches, including utility and benefits for treatment planning and public health. Finally, they highlight the need to embrace cultural and geographic diversity and to deal with stigma and shame currently generated by categorically labeling an individual's personality as “normal” vs. “abnormal” (3, 4).

d'Huart et al. overview the longitudinal research findings that challenge the established diagnostic requirement that PDs must be *stable* over time. The authors show that the balance estimates for PDs and PD symptoms in both adolescents and adults are not that stable. Except for high-risk samples, there is a trend toward symptomatic remission over time. They point out that these findings call the stability of PD into question while arguing in favor of the AMPD and ICD-11 models in which PD features are defined as *relatively* stable over time (5).

Taken together, all three studies appear to highlight features (e.g., what it means to be human, dimensional measurement, and relative stability) that are somewhat taken into account in the more recently published ICD-11 and AMPD frameworks of personality functioning (6).

## ICD-11 and AMPD personality disorders and related traits

Hualparuca-Olivera and Caycho-Rodríguez seek to review the literature on the diagnostic performance of ICD-11 and AMPD measures of PD severity with particular emphasis on clinical sensitivity and specificity. Based on 21 selected studies, the authors conclude that although some empirical support for severity cut-offs exist, these must be taken with caution, since the studies are characterized by substantial deficiencies in methodology (e.g., lack of gold-standard measures, interview data, clinical data, and projective test data), which should therefore be addressed in future studies.

Simon et al. recognize the profound and challenging transition from the traditional types of PD to the new ICD-11 stylistic features of trait domain specifiers. To facilitate this transition, they provide an overview of current studies on associations between PD types and ICD-11 trait domains. Based on nine selected studies from U.S., China, Brazil, Denmark, Spain, Korea, and Canada, the authors propose a cross-walk for translating categorical PD types into ICD-11 trait domains. Consistent with previous observations, the stylistic features of traditional PDs do not seem lost in translation (7). However, the clinical use of trait domains requires a new way of thinking with focus on compositions of trait domains rather than separate trait domains.

## Traditional borderline and narcissistic personality disorders

Wu et al. aims to highlight gaps in the current body of research on borderline PD in primary care. Despite WHO's transition to a fundamentally new diagnostic approach, this review is deemed relevant for future clinical practice as the ICD-11 allows clinicians to code an additional borderline pattern specifier that corresponds to the traditional borderline diagnosis. Emphasis is placed on describing the framework for treatment, identifying psychotherapeutic opportunities, and managing responses to difficult clinical scenarios. The paper particularly emphasizes that borderline PD is prevalent but under-diagnosed and under-treated in primary care, which therefore warrants improved clinical guidelines for these settings. Such guidelines may cover validation of the patient's distress, clear boundaries, communication with the entire treatment team, regular appointments, and psychotherapeutic tools.

di Giacomo et al. reviewed the literature on issues in the empathic attitude of people with narcissistic personality disorder (NPD). Interestingly, they find that individuals characterized by NPD show greater impairment in affective aspects while their cognitive part of empathy appears preserved. As a clinical implication, the authors suggest that by taking advantage of the intact cognitive aspects of empathy, therapeutic improvement of affective aspects may eventually be accomplished. From a contemporary ICD-11 and AMPD perspective, this insight seems relevant for PD patients with personality functioning that is characterized by an unrealistically positive and grandiose self-view as well as those with difficulty recovering from (narcissistic) injuries to self-esteem (8).

## The significance of personality disorder for musculoskeletal disorders

Mental disorders are often comorbid with longstanding health issues that complicate the rehabilitation process (9, 10). From such mind-body perspective, Quirk, Koivumaa-Honkanen, Honkanen et al. and Quirk, Koivumaa-Honkanen, Kavanagh, et al. have contributed with a systematic review protocol and a scoping review (based on 10 reviews and 47 individual analysis) for investigating co-morbidity and associations between PDs and musculoskeletal disorders (e.g., osteoarthritis and fibromyalgia). The authors find noteworthy associations of PD with chronic back/neck/spine conditions, arthritis, fibromyalgia, and reduced bone mineral density, with shared and non-shared risk (and protective) factors, even though they are poorly understood. They conclude that further research is needed to determine if people with PD may be susceptible to bone health issues such as osteoporosis and fragility fractures, and to investigate possible causal mechanisms. In addition, we find it particular relevant that future studies investigate such associations and mechanisms, including global burden of disease, using the ICD-11 and AMPD measures of PD severity and individual trait expressions.

## Author contributions

BB: Conceptualization, Writing—original draft, Writing—review and editing. MB: Writing—review and editing.
